# Unraveling the Nexus Between Ambient Air Pollutants and Cardiovascular Morbidity: Mechanistic Insights and Therapeutic Horizons

**DOI:** 10.7759/cureus.68650

**Published:** 2024-09-04

**Authors:** Vishal Kumar, Chitra Vellapandian

**Affiliations:** 1 Department of Pharmacology, SRM College of Pharmacy, SRM Institute of Science and Technology, Chengalpattu, IND

**Keywords:** cardiovascular disease, atherosclerosis, thrombosis, autonomic nervous system imbalance, endothelial dysfunction, oxidative stress, systemic inflammation, air pollutants

## Abstract

Air pollution poses a significant threat to cardiovascular health, contributing to the development and progression of various heart diseases. This review delves into the intricate relationship between ambient air pollutants and cardiovascular morbidity, elucidating the underlying mechanisms and exploring potential therapeutic approaches. We discuss the major types of air pollutants, including particulate matter (PM), nitrogen dioxide (NO_2_), sulfur dioxide (SO_2_), ozone (O_3_), and carbon monoxide (CO), and their respective roles in exacerbating cardiovascular conditions. The review highlights the key mechanisms by which air pollutants adversely impact the cardiovascular system, including systemic inflammation, oxidative stress, endothelial dysfunction, autonomic nervous system imbalance, and dysregulation of blood coagulation and thrombosis. Vulnerable populations, including children, the elderly, and those with pre-existing health conditions, are disproportionately affected. Air quality regulations aim to mitigate these effects by reducing pollutant levels, with the overall goal of lowering cardiovascular morbidity and improving public health outcomes. Specifically, stringent regulations focus on curbing vehicular emissions and industrial pollutants and promoting cleaner energy sources. Recent data underscore the importance of addressing environmental and behavioral risk factors to prevent the growing global burden of cardiovascular disease. This review synthesizes the mechanistic pathways through which pollutants contribute to cardiovascular damage and highlights the urgent need for early detection strategies and targeted therapies. Improving public health through stricter air quality control measures and raising awareness of the health risks associated with pollution is crucial for mitigating the long-term cardiovascular impacts of air pollution.

## Introduction and background

Air pollution is a major threat to human health, affecting multiple organ systems and leading to a wide range of health problems. It describes the release of different gases, finely split particles, or scattered fluid aerosols into the atmosphere at rates higher than what the ecosystem can naturally absorb or dissipate. These chemicals may accumulate to harmful levels, affecting human health, the economy, and aesthetics. In addition to causing climate change and deteriorating materials, air pollution can significantly impact cardiovascular health.

According to the WHO, cardiovascular diseases (CVDs) accounted for 17.9 million deaths worldwide in 2019, approximately 32% of all fatalities. This makes CVD the leading cause of death worldwide. Of these, 85% of the fatalities were due to heart attacks and strokes. Over 75% of CVD-related fatalities occur in low- and middle-income countries. Approximately 17 million premature deaths (deaths under 70) in 2019 were caused by non-communicable illnesses, with CVDs accounting for 38% of these deaths. Preventing most CVDs is feasible by addressing behavioral and environmental risk factors, including air pollution, poor diets and obesity, physical inactivity, excessive alcohol consumption, and tobacco use. Early detection of CVD is essential to initiate medication and counseling-based therapies.

According to the World Heart Federation's (WHF) recently released World Heart Report 2023, 20.5 million deaths worldwide, approximately one-third of all fatalities, were caused by CVDs in 2021, surpassing the 121 million deaths from CVD that were predicted. CVDs continue to affect over half a billion people worldwide.

Air pollution contributes to the likelihood of developing heart complications. Sustained exposure can lead to conditions such as strokes, heart disease, and lung cancer. Pollutants and fine particulate matter (PM), such as ground-level ozone, can cause organ damage and inflammation by deeply penetrating the bloodstream and lungs. Air pollution aggravates pre-existing respiratory illnesses and raises death rates. It affects the lungs, leading to chronic respiratory conditions such as chronic obstructive pulmonary disease (COPD). Prolonged exposure can damage nerves, the brain, liver, kidneys, and other organs. Additionally, air pollutants may cause allergies, birth defects, and even death.

In this review, we explore the intricate relationship between air pollutants and CVD, elucidate the underlying mechanisms, and discuss therapeutic approaches for early detection and treatment.

## Review

Source and types of air pollutants

Air pollution is acknowledged as a significant contributor to the onset and worsening of CVDs. Various types of air pollutants contribute to cardiovascular health problems, which include the following:

Particulate Matter (PM)

"Particulate matter," often called "particle pollution" or PM, describes a mixture of tiny particles and liquid droplets suspended in the air. A variety of substances can contribute to particle pollution, such as metals, soot, dust or soil particles, organic chemicals, inorganic compounds (such as sodium chloride, ammonium nitrate, and sulfate), acids (such as sulfuric acid), and biological components (such as pollen and mold spores) (Table 1).

**Table 1:** Origins and health impacts of varied particulate matter This table has been made by the authors. Permission is needed before utilizing this table.

Particle	Size	Source	Health Effect
PM_10_ (Coarse Particulate Matter)	≤ 10 micrometers diameter	Dust from roads, construction sites, agricultural activities, industrial processes, and natural sources like pollen and sea spray	Can penetrate the upper respiratory tract and cause dyspnea, coughing, and wheezing, among other breathing problems
PM_2.5_ (Fine Particulate Matter)	≤ 2.5 micrometers diameter	Processes involving combustion (car engines, power plants, home heating, and industrial operations), wildfires, and secondary formation from gaseous pollutants like NOx and sulfur dioxide (SO_2_)	Can cause breathing and cardiovascular issues, such as heart attacks, strokes, and lung disorders, by penetrating deeply into the lungs and into the bloodstream
Ultrafine Particles (UFPs)	0.1 micrometers (100 nanometers) or less in diameter	Combustion engines (especially diesel engines), industrial processes, and certain manufacturing activities. Some are also formed from the reaction of gases	Can penetrate cellular membranes and enter the bloodstream, potentially causing more severe health effects than larger particles, including inflammation, oxidative stress, and adverse cardiovascular and respiratory outcomes
Black Carbon	Specifically, particle pollution refers to soot particles that arise from the incomplete combustion of fossil fuels, biofuels, and biomass	Diesel engines, residential wood burning, industrial emissions, and forest fires	Associated with cancer and is a contributing factor to respiratory and cardiovascular disorders. It also has significant climate impacts by absorbing sunlight and warming the atmosphere
Secondary Organic Aerosols (SOAs)	Typically, within the PM_2.5_ range	Produced in the atmosphere through the chemical interactions of volatile organic compounds (VOCs) that are emitted by vehicles, machinery, and plants	Similar to other fine particles, contributing to respiratory and Cardiovascular diseases
Mineral Dust	Generally, it includes larger particles, but fine particles can also be present	Natural sources such as soil erosion, dust storms, and volcanic eruptions. Human activities like mining and construction also contribute	Can cause respiratory issues and aggravate existing conditions like asthma and bronchitis
Sea Salt Particles	Can range from coarse to fine particles	Formed from the ocean's spray when the wind blows over the surface of the sea	Generally considered less harmful compared to other types of PM but can still cause respiratory issues in sensitive individuals

Nitrogen Dioxide (NO_2_)

NO_2_ contributes to CVDs through several mechanisms, including endothelial dysfunction, oxidative stress, and inflammation. Exposure to NO_2_ impairs endothelial function, which is essential for maintaining vascular health, leading to reduced nitric oxide (NO) availability and increased vascular resistance. Additionally, NO_2_ exposure induces the production of reactive oxygen species (ROS), resulting in oxidative stress that damages endothelial cells and promotes inflammatory responses, contributing to conditions such as atherosclerosis. Experimental research has shown that repeated NO_2_ exposure can lead to mitochondrial dysfunction and alterations in coronary vascular reactivity, further exacerbating cardiovascular issues. In terms of atherosclerosis development, studies indicate that chronic NO_2_ exposure accelerates the accumulation of lipids in arterial walls and enhances inflammatory processes, leading to plaque formation. This is associated with decreased myocardial perfusion and increased ROS production, creating a vicious cycle of cardiovascular impairment.

Epidemiological evidence corroborates these findings. According to a Beijing-based study, for each daily mean rise of 10 μg/m^3^ in NO_2_, there is an increase in cardiovascular mortality overall of 1.89%, cerebrovascular mortality of 2.07%, and ischemic heart disease mortality of 1.95%. These effects were more pronounced in districts with larger populations, greater coal consumption, and higher numbers of vehicles [1].

Adverse cardiac remodeling in individuals with dilated cardiomyopathy has also been associated with chronic exposure to NO_2_, with a higher risk observed in women. Following NO_2_ exposure, patients with pre-existing cardiac disease may experience symptoms including weariness, lightheadedness, shortness of breath, and chest discomfort.

Sulfur Dioxide (SO_2_)

SO_2_ is a significant air pollutant that exacerbates CVDs through multiple mechanisms. It induces systemic inflammation and oxidative stress, leading to endothelial dysfunction and impaired vascular function. This damage decreases NO availability, promotes vasoconstriction, and accelerates CVD progression.

Both short-term and long-term exposure to SO_2_ can have severe cardiovascular effects. Brief exposure can cause elevated blood pressure and heart rate, while prolonged exposure is linked to sustained hypertension, accelerated atherosclerosis, and increased risks of heart attack and stroke. Chronic exposure leads to persistent endothelial dysfunction and systemic inflammation, which can reduce left ventricular pressure and heart rate, increase coronary flow, and ultimately decrease life expectancy.

Histopathological changes associated with SO_2_ exposure include inflammatory cell infiltration, myocyte damage, edema, myocardial infarction, fiber atrophy, and necrosis. These changes contribute to impaired cardiac function and higher risks of heart failure. Additionally, the combination of aerobic exercise with SO_2_ exposure further exacerbates cardiovascular effects, increasing left ventricular end-diastolic pressure, angiotensin II concentration, and angiotensin-converting enzyme (ACE) activity. Overall, SO_2_ exposure significantly worsens cardiovascular health through increased oxidative stress, reduced ATPase activity, structural remodeling of the heart, and activation of the renin-angiotensin system.

Ozone (O_3_)

O_3_ is produced as a secondary air pollutant when volatile organic compounds (VOCs) and nitrogen oxides (NOx) undergo chemical reactions under sunlight. VOC originates from sources such as motor vehicles, power plants, industrial facilities, and the combustion of biomass and fossil fuels.

O_3_ exposure can lead to cardiovascular effects through several interconnected mechanisms. It increases oxidative stress and systemic inflammation, which can induce endothelial dysfunction, heightened vascular reactivity, and alterations in ANS function. These changes promote atherosclerosis by enhancing platelet activation and thrombus formation and impairing blood flow regulation. Collectively, these mechanisms underscore the cardiovascular risks associated with O_3_ exposure, highlighting its potential role in exacerbating existing conditions and increasing the likelihood of acute events such as heart attacks and strokes.

Elevated O_3_ exposure has been linked to a rise in hospital admissions for heart-related conditions such as heart attacks, strokes, and heart failure. Every 10 µg/m^3^ increase in the two-day average eight-hour maximum O_3_ concentration resulted in an increase of 0.40% and 0.75%, respectively, in hospital admissions for stroke and acute myocardial infarction. Hospital admissions for heart-related illnesses increased significantly when O_3_ levels were above the WHO limit of 100 µg/m^3^; the range for stroke was 3.38%, and for acute myocardial infarction was 6.52% [2]. O_3_ exposure can worsen pre-existing CVDs and promote the emergence of new illnesses.

Carbon Monoxide (CO)

CO is a colorless, odorless gas produced by the incomplete combustion of carbon-based fuels, with major sources including motor vehicles, power plants, wildfires, and faulty appliances. Brief exposure to high levels of CO has been linked to increased hospital admissions for CVD and heart failure-related mortality. CO worsens pre-existing CVD by reducing the blood's oxygen-carrying capacity, as it binds to hemoglobin 200-250 times more strongly than oxygen, forming carboxyhemoglobin. This leads to insufficient oxygen delivery to organs, including the heart, causing myocardial ischemia, arrhythmias, and potentially heart failure [3].

CO exposure also contributes to oxidative stress and mitochondrial dysfunction, generating ROS that damage cells and impair energy production, further exacerbating cardiovascular dysfunction. Acute antioxidant treatment has shown the potential to reverse these effects in animal studies. Symptoms in CVD patients exposed to CO include chest pain, shortness of breath, dizziness, and confusion.

Mechanism

Systemic Inflammation

Systemic inflammation is a critical mechanism by which air pollution contributes to CVD. This process involves the body's widespread inflammatory response to harmful pollutants, which can lead to various pathological changes in the cardiovascular system. Systemic inflammation is a chronic, low-grade inflammatory state affecting the entire body. It is marked by elevated levels of acute-phase proteins and pro-inflammatory cytokines in the bloodstream. Unlike localized inflammation, which is restricted to a specific tissue or organ, systemic inflammation has widespread effects and can impact multiple systems, including the cardiovascular system.

Inhaled pollutants reach the lungs and cause local inflammation. Due to its small size, PM can penetrate deep into the alveoli. Pollutants in the alveoli activate alveolar macrophages and other immune cells. These cells produce inflammatory signaling molecules such as IL-1B, IL-6, and TNF-a, as well as ROS. The cytokines produced in the lungs enter the systemic circulation, spreading the inflammatory response beyond the lungs. Inflammatory signals can stimulate the bone marrow to release more inflammatory cells, such as monocytes, into the bloodstream. Some air pollutants can directly enter the bloodstream and interact with endothelial cells lining the blood vessels, causing oxidative stress and endothelial damage. The systemic inflammatory response also plays a role in endothelial dysfunction, marked by decreased availability of NO. NO plays a crucial role in maintaining vascular homeostasis by promoting vasodilation, inhibiting platelet aggregation, and preventing leukocyte adhesion to the endothelium [4]. Monocytes are drawn to the endothelium by inflammatory cytokines and endothelial dysfunction, where they undergo macrophage differentiation. After consuming oxidized low-density lipoprotein (LDL) particles, these macrophages develop into foam cells and start the development of fatty streaks, which are precursors to atherosclerosis. Persistent inflammation contributes to the advancement of these initial fatty streaks into more intricate atherosclerotic plaques.

Furthermore, inflammatory cytokines can break down the fibrous layer of plaques, increasing the likelihood of a rupture. A thrombus, or blood clot, forms when an atherosclerotic plaque bursts, exposing thrombogenic material to circulation. This thrombus can occlude a coronary artery, causing myocardial infarction (heart attack), or a cerebral artery, leading to a stroke. Systemic inflammation also promotes a hypercoagulable state, further increasing the risk of thrombosis.

Oxidative Stress

When PM, especially fine particles such as PM_2.5_, is inhaled, it can directly generate ROS within the respiratory tract. The surface of PM can contain metals and other reactive compounds that catalyze ROS formation. Ozone, a common air pollutant, is a strong oxidizing agent. When inhaled, it can directly interact with the lining of the respiratory tract, leading to the formation of ROS. This direct generation of ROS occurs primarily in the lungs, where these pollutants are first encountered.

Pollutants can activate NADPH oxidase, an enzyme complex found in cell membranes that produces superoxide, a type of ROS, from oxygen. This activation occurs as part of the body's immune response to the presence of foreign particles. Pollutants can also impair mitochondrial function, leading to increased ROS production. Mitochondria are essential for cellular energy production, and their dysfunction can lead to the release of electrons that react with oxygen, generating ROS.

ROS can interact with lipids in cell membranes, causing lipid peroxidation. This process damages the integrity and function of the membranes, leading to cellular dysfunction. Additionally, ROS can oxidize proteins, altering their structure and function, which impairs enzyme activity, disrupts signaling pathways, and compromises the structural integrity of cells. Moreover, ROS can induce mutations and strand breaks in DNA, resulting in genomic instability that can contribute to cancer and other diseases.

The oxidative damage to lipids, proteins, and DNA results in impaired cellular functions. This can affect cell signaling, metabolism, and overall cell health. Severe oxidative damage can trigger apoptosis, or programmed cell death, as cells are unable to repair the damage and maintain normal function.

Nitric oxide is a critical molecule for vascular health, as it promotes vasodilation and helps maintain blood vessel homeostasis. ROS can react with NO to form peroxynitrite, a reactive nitrogen species that reduces NO availability [5]. The reduction in NO availability impairs endothelial function, as NO is essential for relaxing blood vessels and maintaining proper vascular tone. This leads to endothelial dysfunction, a precursor to atherosclerosis and other CVDs [4].

Endothelial Dysfunction

The endothelium, a thin layer of cells lining the inside surface of blood vessels, is crucial for maintaining vascular health by regulating blood flow, vessel tone, and the balance between coagulation and fibrinolysis. Endothelial dysfunction is a key mechanism through which air pollution contributes to CVD.

Particularly fine PM (PM_2.5_) exposure has been strongly linked with the growth and development of CVD through the induction of endothelial dysfunction. The primary mechanism involves an imbalance between vasodilator and vasoconstrictor factors in the vasculature, leading to impaired vascular homeostasis. A key mediator is the endothelium-derived vasoconstrictor peptide endothelin-1 (ET-1). Air pollution exposure increases the synthesis and activity of ET-1 and its receptors, which leads to vasoconstriction and impaired vasodilation. In addition, air pollution induces systemic oxidative stress and inflammation within the vasculature. This leads to decreased bioavailability of NO, a potent vasodilator derived from endothelial nitric oxide synthase (eNOS). The imbalance between increased ET-1 and decreased NO contributes to endothelial dysfunction [6]. Furthermore, air pollution can directly damage vascular endothelial cells, impairing endothelial barrier function and increased permeability. This promotes atherosclerosis development and increases the risk of thrombosis and cardiovascular events.

Chronic exposure to air pollution has been associated with decreased amounts of endothelial progenitor cells (EPCs), which are essential for maintaining vascular homeostasis and facilitating endothelium repair. Due to continuous mobilization and fatigue, chronic exposure causes cumulative harm to the bone marrow or circulating EPCs, reduced EPC release, and decreased availability of EPCs.

ANS Imbalance

Air pollution exposure, especially fine PM (PM_2.5_), can lead to an imbalance in the ANS, which is a key mechanism contributing to CVD. The ANS regulates vital signs, including blood pressure, heart rate, and breathing rate. It consists of two main branches: the sympathetic nervous system (SNS), which increases heart rate and blood pressure to prime the body for "fight or flight" reactions, and the parasympathetic nervous system (PNS), which lowers heart rate and promotes healing processes to enable "rest and digest" activities.

When inhaled, air pollutants can affect the cardiovascular system both directly and indirectly. Inhalation of air pollutants can stimulate the SNS. This activation may be mediated by (1) pulmonary reflexes (pollutants irritate the airways, triggering reflexes that activate the SNS) and (2) systemic inflammation (pollutants cause inflammation in the lungs, releasing pro-inflammatory cytokines into the bloodstream; these cytokines can activate the SNS). Simultaneously, air pollutants can suppress the activity of the PNS, reducing its ability to counterbalance the SNS. This suppression can be due to oxidative stress (pollutants induce oxidative stress, damaging the cells and tissues involved in PNS regulation) and inflammatory mediators (the inflammatory response can interfere with PNS signaling pathways). The combined effect of heightened SNS activity and reduced PNS activity leads to ANS imbalance. This imbalance manifests as increased heart rate and blood pressure variability, which are risk factors for cardiovascular events.

An overactive SNS increases heart rate and blood pressure, putting additional strain on the cardiovascular system. Chronic elevation of these parameters can lead to hypertension and increase the risk of heart attacks and strokes. Additionally, pollutant-induced oxidative stress and inflammation can damage the endothelium, the blood vessels' inner lining. Endothelial dysfunction impairs the vessel's ability to dilate and constrict properly, leading to atherosclerosis (hardening of the arteries). ANS imbalance increases the risk of arrhythmias (irregular heartbeats). Overactivation of SNS and suppression of PNS disrupt the electrical stability of the heart, potentially causing life-threatening arrhythmias. Chronic inflammation, driven by persistent exposure to pollutants, contributes to the development and progression of CVD. Inflammation promotes plaque formation in the arteries and can lead to plaque rupture, causing heart attacks.

Blood Coagulation and Thrombosis

Blood coagulation is the process by which blood forms clots to prevent excessive bleeding. Thrombosis is the occurrence of a blood clot inside a blood vessel, potentially obstructing blood flow within the circulatory system. Both processes are crucial for maintaining vascular integrity but can lead to CVD when dysregulated.

When air pollutants enter the respiratory system upon inhalation, they can have systemic effects. Pollutants induce inflammation in the respiratory tract, prompting the release of pro-inflammatory cytokines and ROS. Due to their ability to penetrate the circulation, these inflammatory chemicals can lead to oxidative stress and systemic inflammation, two major factors that promote thrombosis and coagulation. The endothelium, or inner lining of blood arteries, can sustain damage from oxidative stress. When endothelial cells are damaged, they express adhesion molecules and release tissue factor (TF), which triggers the coagulation cascade. Pollutants can directly or indirectly activate platelets, making them more likely to adhere to each other and the vessel wall, forming clots [7,8]. Systemic inflammation leads to an increase in the production of clotting factors such as fibrinogen and von Willebrand factor, promoting a procoagulant state. The process that breaks down clots (fibrinolysis) is impaired due to increased levels of plasminogen activator inhibitor-1 (PAI-1), which stops the disintegration of fibrin clots.

Air pollution can increase the tendency for clot formation (hypercoagulability), leading to thrombosis. This can occur in both arteries and veins, leading to conditions such as deep vein thrombosis (clots in the leg's deep veins), pulmonary embolism (clots that travel to the lungs, which can be life-threatening), and arterial thrombosis (clots in the arteries, leading to myocardial infarction (heart attack) or stroke).

Chronic inflammation and oxidative stress due to air pollution accelerate the progression of atherosclerosis. Plaque rupture can trigger the coagulation cascade, leading to the creation of a thrombus that can block blood flow. The combination of increased clotting factors, platelet activation, and endothelial dysfunction increases the risk of coronary thrombosis, where a clot forms in the coronary arteries. This may prevent blood from reaching the heart muscle, which might result in a heart attack.

Thrombi can also form in the cerebral arteries, leading to ischemic stroke. Systemic inflammation and a procoagulant state heighten the risk of stroke in individuals exposed to elevated levels of air pollution.

Vulnerable population

Vulnerable populations particularly affected by air pollution-related CVD include elderly individuals whose susceptibility stems from age-related physiological decline and the presence of pre-existing health conditions. As people age, their cardiovascular and respiratory systems weaken, making it harder for their bodies to cope with the inflammatory and oxidative stress induced by air pollutants. This can accelerate the progression of cardiovascular conditions such as atherosclerosis and heart failure.

In children, the developing cardiovascular system is susceptible to pollutants such as PM and ozone. Exposure during critical periods of growth can result in structural changes to the heart and blood vessels, potentially leading to long-term health consequences such as hypertension and an elevated risk of CVD later in life.

People with pre-existing medical conditions, such as heart disease or hypertension, are more vulnerable to air pollution as their already compromised cardiovascular systems are further strained by the additional oxidative stress and inflammation caused by pollutants. For individuals with respiratory conditions such as asthma and COPD, exposure to air pollution can worsen symptoms and lead to increased cardiovascular stress due to reduced oxygenation, which places an extra burden on the heart.

Low socioeconomic status (SES) communities often reside in areas with higher levels of pollution due to industrial proximity or dense traffic. These communities also have limited access to healthcare and resources for managing pollution exposure, which compounds their vulnerability to air pollution-related cardiovascular issues. Moreover, outdoor workers, such as construction workers, farmers, and traffic police, face prolonged exposure to pollutants, elevating their risk of CVD. Additionally, some minority groups are disproportionately affected, as they are more likely to live in polluted areas and experience barriers to accessing healthcare, heightening their susceptibility to cardiovascular impacts from air pollution.

Public health strategies need to focus on protecting vulnerable groups through targeted interventions such as stricter air quality regulations in high-risk areas, health education, and the provision of resources such as air purifiers. Stricter air quality regulations should include the establishment of low-emission zones (LEZs) to limit access for high-polluting vehicles, enhanced emission standards for both new and existing vehicles, and stringent controls on industrial emissions through advanced filtration technologies. Promoting public transportation and active travel, alongside urban planning that incorporates green spaces, can further reduce pollution exposure. Enhanced monitoring and surveillance of air quality and cardiovascular health trends in vulnerable populations can help in the early detection and prevention of adverse health outcomes. Additionally, public awareness campaigns about the health impacts of air pollution are essential for empowering communities. Regular monitoring and reporting of air quality data will enhance transparency, while health impact assessments for new developments can ensure that air quality considerations are integrated into decision-making processes. Mitigating the effects of air pollution on cardiovascular health requires ensuring equal access to healthcare and preventative services for all populations, especially the most vulnerable. Collectively, these measures aim to significantly improve air quality and protect vulnerable populations from adverse health effects.

Lifestyle habits that increase CVD susceptibility

Air pollution is a major risk factor for CVD, and certain lifestyle factors can further amplify its harmful effects. Understanding the interplay between these factors and pollution can help develop effective strategies to mitigate cardiovascular risks.

Physical Inactivity

Lack of regular physical activity is a major risk factor for CVD. Sedentary behavior and insufficient exercise can have several negative health impacts that raise the risk of CVD. Physical inactivity is associated with weight gain, high blood pressure, elevated cholesterol levels, and other metabolic issues. According to studies, compared to more active individuals, people who are less physically fit and active have a 30%-50% increased risk of high blood pressure. Additionally, a 24% higher risk of coronary heart disease, a 16% higher risk of stroke, and a 42% higher risk of type 2 diabetes have all been associated with inactivity [9]. Regular physical exercise can aid in the reduction of these conditions, which pose significant risks for CVD. Maintaining an active lifestyle and engaging in the recommended 150 minutes of moderate exercise per week can help reduce the risk of cardiovascular events and death.

Diet and Nutrition

The WHO states that changing to a healthier diet can dramatically lower the chance of getting CVD. A diet that is high in cholesterol, trans fats, saturated fats, and sodium significantly contributes to the development of CVD. These dietary components can cause plaque to accumulate in the arteries, leading to their narrowing and hardening, which in turn increases resistance to blood flow. This condition, known as atherosclerosis, contributes to high blood pressure as the heart must exert more effort to pump blood through the narrowed vessels. High cholesterol levels, particularly LDL (bad) cholesterol, can also occur due to an unhealthy diet. Additionally, weight gain and fatness are common side effects of a diet heavy in these harmful ingredients, and they are also independent risk factors for CVD.

Alcohol Consumption

Heavy or binge drinking is strongly linked with an increased risk of several CVDs, e.g., hypertension, stroke, and heart attack. Excessive alcohol intake can lead to hypertension, with studies showing that binge drinking can increase systolic and diastolic blood pressure by 4-7 mmHg and 4-6 mmHg, respectively. This increase in blood pressure is concerning, as even small rises in blood pressure are linked to higher risks of stroke and coronary artery disease (CAD). In addition, chronic heavy drinking may be a factor in the development of cardiomyopathy, a disorder that weakens and damages the cardiac muscle. Excessive alcohol use has also been shown to negatively interact with certain medications used to treat CVD, potentially reducing their effectiveness.

Stress and Mental Health

Persistent stress can have serious negative consequences on the cardiovascular system, which can accelerate the onset and course of CVD. Extended exposure to stress hormones such as catecholamines and cortisol can raise blood pressure because these chemicals narrow blood vessels and make the heart beat faster. Prolonged stress also makes the body more inflammatory, which can damage blood vessel walls and raise the risk of atherosclerosis by clogging the arteries with plaque. Additionally, stress can lead to unhealthy habits, such as smoking and high cholesterol and poor diet, which exacerbate CVD risk factors such as diabetes, obesity, and inactivity. Chronic stress has also been linked to cardiac arrhythmias, endothelial dysfunction, and a condition known as "broken heart syndrome" that can temporarily weaken the heart muscle.

Overweight/Obesity

The risk of having numerous CVDs risk factors is greatly increased when one is overweight or obese, particularly if there is extra fat around the abdomen. Type 2 diabetes, high blood pressure, and high cholesterol are all closely linked to being overweight. A meta-analysis involving more than 300,000 adults revealed that a body mass index (BMI) within the overweight or obese categories is linked to a higher risk of developing CAD. Being overweight, especially in the belly area, can cause metabolic irregularities, inflammation, and insulin resistance, all of which, over time, harm the cardiovascular system. Studies have demonstrated that altering one's lifestyle to even slightly lower body weight can improve these cardiometabolic risk factors and lower the chance of getting CVD. Maintaining a healthy weight with a balanced diet and regular exercise is the key to preventing cardiovascular issues linked to obesity.

Diabetes

Diabetes significantly increases the risk of heart disease, stroke, and heart attack, especially when it is not properly controlled. Diabetes-related persistently elevated blood sugar levels can damage blood vessels and the heart over time, resulting in a variety of cardiovascular problems. Individuals with diabetes are almost twice as likely to suffer from heart disease and stroke as those without the condition. Atherosclerosis, or the buildup of cholesterol plaque in the arteries, is a condition that can result from poorly controlled diabetes. Blood flow to the heart and brain is impeded by this disorder, which causes blood vessels to stiffen and constrict. This raises the risk of a heart attack and stroke. Diabetes also increases the risk of other CVDs, such as obesity, high blood pressure, and excessive cholesterol levels, all of which increase the risk of heart disease. Reduced cardiovascular consequences from diabetes are mostly dependent on proper blood sugar, blood pressure, and cholesterol management achieved by medication and lifestyle modifications.

Smoking

Smoking is an important preventable risk factor for CVD, with smokers having a two to four times higher risk of heart disease than non-smokers. There are several ways in which smoking cigarettes harms the cardiovascular system, including promoting atherosclerosis, increasing blood pressure, and reducing oxygen supply to the heart. Smoking causes inflammation and endothelial dysfunction, leading to plaque buildup in the arteries. Additionally, the capacity of red blood cells to transport oxygen is decreased by carbon monoxide in cigarette smoke, depriving the heart of oxygen necessary for life. Because of these harmful consequences, smoking greatly increases the risk of heart attack, stroke, and coronary heart disease. The good news is that stopping smoking can significantly lower this risk over time, and the longer a person stays smoke-free, the more advantages they will experience.

Cardiovascular consequences of e-cigarette use and cannabis smoking in emerging adulthood

Cardiovascular Risks of E-cigarettes/Vaping in Young Adults

E-cigarette use among youth is associated with an elevated risk of CVD. Young users of e-cigarettes may experience increased arterial stiffness, higher blood pressure, elevated oxidative stress, and inflammation. Starting vaping in young adulthood could also lead to lifelong nicotine addiction, significantly increasing the likelihood of developing CVD later in life. E-cigarettes adversely impact the cardiovascular system, with nicotine and other chemicals in the aerosol activating inflammatory processes and having a direct role in the emergence of CVD and acute cardiovascular events. E-cigarette use leads to rapid deterioration of vascular function, akin to traditional cigarette smoking. It also results in a shift in the balance of the ANS towards sympathetic predominance, a factor linked to heightened cardiac risk. Cross-sectional analysis has shown that daily e-cigarette use is linked to higher odds of experiencing a myocardial infarction, with the effect approaching that of conventional cigarette smoking. Even exclusive e-cigarette users had a risk of developing CVD that did not differ from non-users, while dual users of e-cigarettes and combustible cigarettes had a significantly increased risk [10,11].

Cardiovascular Risks of Cannabis Smoking in Young Adults

Cannabis use is linked to an elevated risk of stroke and myocardial infarction in young adults, with greater frequency of use associated with higher odds of these events. Frequent cannabis smoking may significantly increase the risk of heart attack by 25% and stroke by 42% compared to non-use, even with less frequent weekly use. Approximately 75% of cannabis users report smoking the drug, which releases toxins similar to those found in tobacco smoke when burned. Smoking cannabis may pose additional cardiovascular risks from inhaling PM. The cardiovascular risks appear to be independent of the effects of tobacco use. Although the exact pathways linking cannabis use to heart disease are not entirely understood, they most likely involve endothelial dysfunction, oxidative stress, altered platelet function, hemodynamic effects, and atherosclerosis. Among young adults vulnerable to early CVD, cannabis use has been associated with conditions such as coronary heart disease, myocardial infarction, stroke, and composite outcomes, even after adjusting for other variables [12,13].

Exercise and workout strategies to reduce CVD

Exercise and workout strategies are essential for reducing CVD risk, and incorporating various forms of physical activity can significantly enhance heart health. Aerobic workouts increase breathing and heart rate, which enhances the cardiovascular system's oxygen transport efficiency. These exercises include walking, jogging, cycling, swimming, and dancing. Weekly aerobic exercise recommendations call for 150 minutes of moderate-intensity or 75 minutes of high-intensity exercise. Strength training, including weightlifting, bodyweight workouts, and resistance band exercises, also plays a crucial role in improving insulin sensitivity, reducing fat mass, and increasing muscle mass and overall body composition. It is recommended to work on all main muscle groups at least twice a week. High-intensity interval training (HIIT), involving short, vigorous bouts of exercise interspersed with brief rest periods, effectively enhances cardiovascular fitness and reduces CVD risk factors. Incorporating HIIT sessions of 20-30 minutes, three to five times per week, can yield substantial benefits.

Flexibility and balance exercises, such as yoga, pilates, and stretching routines, complement cardiovascular workouts by enhancing overall physical function and reducing injury risks. Integrating these exercises two to three times per week is beneficial. Beyond formal exercise, increasing daily physical activity through lifestyle integration, such as opting for stairs over elevators, walking or cycling to work, active commuting, gardening, and household chores, promotes cardiovascular fitness and a healthier lifestyle. Monitoring progress is vital for staying motivated and achieving fitness goals. Gradually increasing workout intensity and duration, using fitness trackers, maintaining an exercise journal, and setting SMART goals (Specific, Measurable, Achievable, Relevant, and Time-bound) can optimize your fitness routine for continuous improvement.

A balanced diet full of fruits, vegetables, whole grains, lean meats, and healthy fats, together with regular exercise, promotes the cardiovascular system even more. Emphasizing reduced intake of saturated and trans fats, moderating salt and sugar consumption, and ensuring adequate hydration complements fitness efforts and fosters overall well-being. Regular health evaluations with a healthcare provider are crucial for monitoring cardiovascular health and ensuring exercise regimen safety. These consultations allow ongoing assessment and necessary adjustments to the fitness program, optimizing cardiovascular well-being and addressing potential health concerns promptly. Integrating these strategies creates a comprehensive approach to reducing CVD risk and enhancing overall health.

Recent advancements in the early detection and treatment of CVD

**Figure 1:**
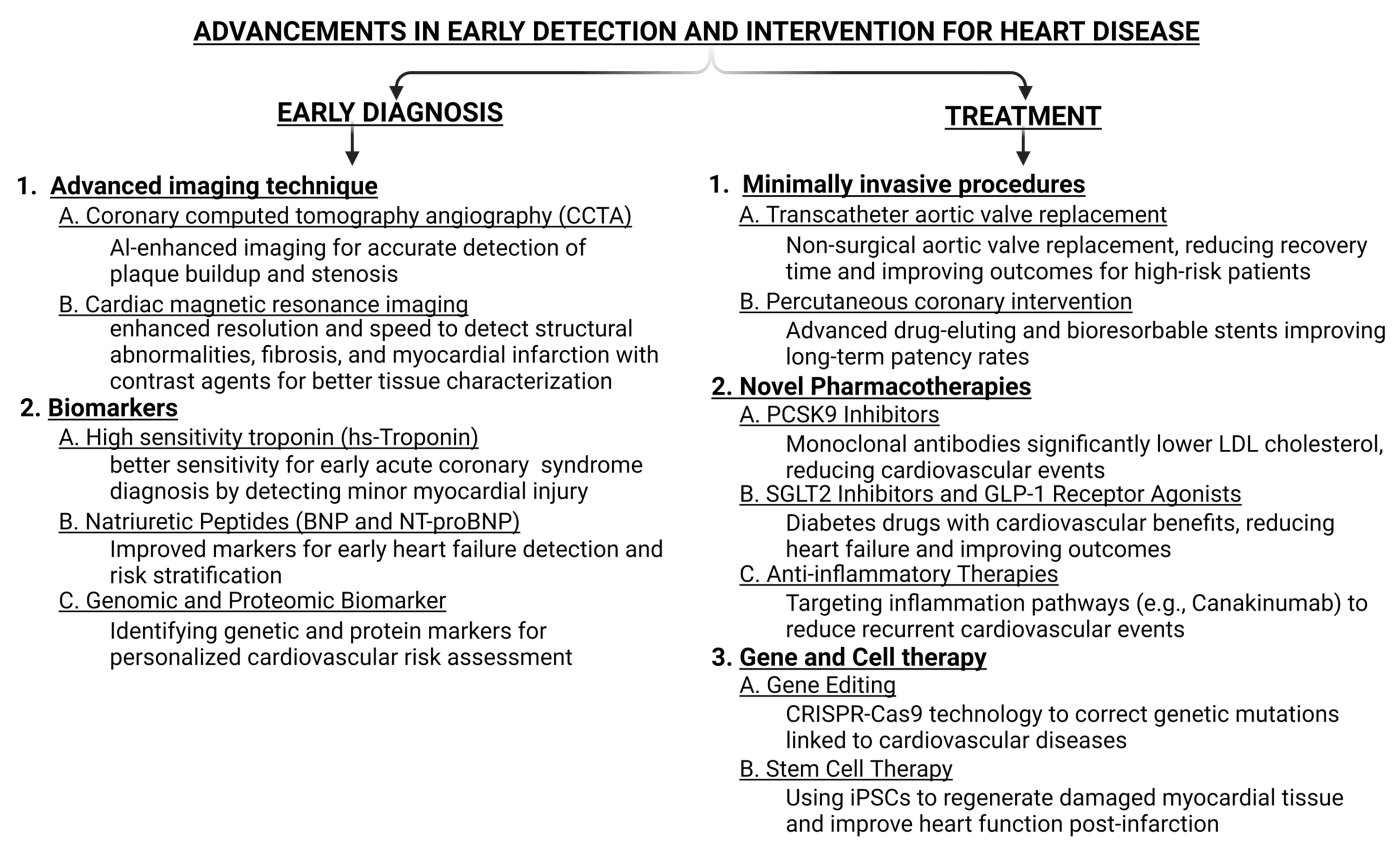
Types of diagnostic and treatment therapy The figure is the original illustration of the author. It is created using BioRender.com. Image credit: Vishal Kumar.

Recent innovations in CVD detection and treatment emphasize advanced diagnostic methods and therapeutic strategies, significantly enhancing early identification and management of heart conditions, ultimately improving patient outcomes, and reducing healthcare costs (Figure 1).

Coronary Computed Tomography Angiography (CCTA)

CCTA is a non-invasive imaging technique used to create detailed images of the coronary arteries, which supply blood to the heart muscle. It employs computed tomography (CT) technology and contrast material to help accurately detect CAD. CCTA involves having the patient receive an intravenous injection of a contrast dye, which highlights the coronary arteries on the CT images. The patient lies on a table that slides into the CT scanner. During the scan, X-ray beams rotate around the patient to capture multiple images of the heart. Advanced software reconstructs the captured images into detailed 3D views of the coronary arteries.

CCTA enables precise detection of plaque buildup, stenosis (narrowing), and other abnormalities. Unlike traditional coronary angiography, which requires catheter insertion, CCTA is non-invasive, reducing the risk of complications and recovery time. CCTA can identify CAD in its early stages, often before symptoms manifest, enabling prompt intervention and effective management [14].

Artificial Intelligence (AI) algorithms analyze the CCTA images to identify early signs of plaque buildup and stenosis. These algorithms can detect minute details that may be missed by the human eye. AI can assess the composition and severity of plaques, providing valuable information about the patient's risk of cardiovascular events. It can differentiate between stable and unstable plaques, aiding in risk stratification. AI speeds up the image analysis process, providing faster and more accurate results. This efficiency helps in quicker diagnosis and decision-making for treatment plans.

The combination of high-resolution imaging and AI analysis enhances the diagnostic accuracy of CCTA, leading to better detection and characterization of CAD. Detailed insights from AI-assisted CCTA enable personalized treatment plans based on the specific characteristics of the patient's coronary arteries and plaque composition. Early detection of CAD through CCTA allows for proactive management, including lifestyle changes, medications, and interventions to prevent progression and reduce the risk of heart attacks.

Cardiac Magnetic Resonance Imaging (Cardiac MRI)

Cardiac MRI has emerged as a potent diagnostic instrument for the evaluation of various cardiac conditions, including structural abnormalities, fibrosis, and myocardial infarction. Recent advancements in cardiac MRI technology have led to improved resolution, speed, and tissue characterization capabilities, enhancing its clinical utility. Cardiac MRI has undergone significant technological advancements, resulting in improved spatial and temporal resolution. These improvements allow for better visualization and assessment of cardiac structures, including the myocardium, valves, and blood vessels. The increased speed of cardiac MRI scans has also reduced the need for breath-holding, making the procedure more comfortable for patients and reducing motion artifacts.

Cardiac MRI's high-resolution imaging capabilities enable the detailed assessment of cardiac anatomy and the identification of various structural abnormalities, such as congenital heart defects, cardiomyopathies, valvular diseases, and pericardial conditions. The ability to visualize these structural abnormalities with high accuracy helps clinicians in the diagnosis, treatment planning, and monitoring of cardiac diseases.

Cardiac MRI, with the use of contrast agents, provides excellent tissue characterization capabilities. This allows for the detection and quantification of myocardial fibrosis, which is a hallmark of various cardiac conditions, including ischemic heart disease, cardiomyopathies, and myocarditis. Furthermore, cardiac MRI can accurately identify and assess myocardial infarction. By employing late gadolinium enhancement (LGE) imaging, areas of scarred or non-viable myocardium can be visualized, allowing clinicians to evaluate the severity of the infarction and make informed treatment decisions.

The use of contrast agents, such as gadolinium-based compounds, enhances the tissue characterization capabilities of cardiac MRI. These contrast agents accumulate in areas of fibrosis, inflammation, or myocardial injury, allowing for the differentiation of healthy and diseased myocardium. This improved tissue characterization aids in the diagnosis and management of various cardiac conditions [15].

High-Sensitivity Troponin (hs-Troponin)

Troponin is a well-established biomarker for the diagnosis of myocardial injury, and the development of hs-troponin assays has significantly enhanced its clinical utility, particularly in the context of acute coronary syndrome (ACS).

Conventional troponin assays have been widely used in the past to detect myocardial infarction and other forms of acute myocardial injury. However, these assays have a relatively high limit of detection, which means they can only reliably detect larger amounts of troponin released into the bloodstream, typically associated with significant myocardial damage. The hs-troponin assays have been developed to overcome the limitations of conventional troponin assays. These new assays have a significantly lower detection limit, allowing for the detection of much smaller amounts of troponin in the blood. The increased sensitivity of hs-troponin assays enables the detection of minor myocardial injury, which may not have been captured by conventional troponin assays. This is particularly relevant in the context of ACS, where prior diagnosis and prompt treatment are crucial for improving patient outcomes. The ability of hs-troponin assays to detect minor myocardial injury allows for the earlier diagnosis of ACS, including myocardial infarction. This is because even small amounts of troponin released into the bloodstream can be detected, indicating the presence of ongoing myocardial damage [16].

Natriuretic Peptides (B-type Natriuretic Peptide (BNP) and N-terminal Pro-B-Type Natriuretic Peptide (NT-proBNP))

Natriuretic peptides, including BNP and NT-proBNP, have emerged as valuable biomarkers in the diagnosis, management, and risk stratification of various cardiac conditions, particularly heart failure. Natriuretic peptides are released from the myocardium, mainly from the ventricles, in response to elevated wall stress and pressure overload. This release occurs in conditions such as heart failure, myocardial infarction, and other forms of cardiac dysfunction. The peptides help the body manage increased cardiac workload by promoting vasodilation, natriuresis (increased sodium and water excretion), and suppression of the renin-angiotensin-aldosterone system (RAAS).

Natriuretic peptides, particularly NT-proBNP, have demonstrated superior specificity and sensitivity in the early detection of heart failure, even in asymptomatic or mildly symptomatic patients. This allows for earlier diagnosis and implementation of appropriate treatment strategies, potentially improving patient outcomes.

Natriuretic peptides, including BNP and NT-proBNP, have emerged as valuable biomarkers for risk stratification in various cardiac conditions. In heart failure, natriuretic peptide levels correlate with disease severity, allowing clinicians to guide treatment decisions and monitor disease progression. For patients with ACS, elevated natriuretic peptides indicate the presence of myocardial injury and can predict adverse outcomes such as mortality and heart failure. In valvular heart disease, natriuretic peptide measurements can help assess the severity of the condition and inform treatment strategies. Additionally, elevated natriuretic peptides are linked to a higher risk of atrial fibrillation and other arrhythmias. Overall, the ability of BNP and NT-proBNP to provide prognostic information across a range of cardiac disorders has made them essential tools for risk stratification and clinical decision-making [17].

Genomic and Proteomic Biomarkers

Genomic and proteomic biomarkers play an essential role in personalized cardiovascular risk assessment. These biomarkers provide valuable insights into an individual's genetic predisposition and protein expression patterns, which can help identify those at higher risk of developing CVDs. Genomic biomarkers involve the identification of specific genetic variants or mutations that are linked to an elevated risk of CVD. These genetic markers can be detected through various genetic testing methods, such as genome-wide association studies (GWAS) and whole-genome sequencing.

Some examples of genomic biomarkers for cardiovascular risk include the following: certain SNPs in genes involved in lipid metabolism, inflammation, and vascular function have been linked to a higher risk of conditions such as myocardial infarction, stroke, and CAD. Variations in genes such as PCSK9, LDLR, and APOE can influence an individual's LDL cholesterol levels and, consequently, their risk of developing atherosclerosis. Genetic variants in genes related to the coagulation cascade, such as factor V Leiden and prothrombin gene mutations, can elevate the risk of thrombotic events, including pulmonary embolism and deep vein thrombosis.

Proteomic biomarkers focus on the identification and quantification of specific proteins or protein patterns that are associated with CVD risk. These biomarkers can be measured in various biological samples, such as blood, urine, or tissue. Proteomic biomarkers that are useful for assessing cardiovascular risk include those that involve the inflammatory response, such as interleukin-6, TNF-α, and C-reactive protein, which have been associated with a higher risk of plaque buildup and cardiovascular events. Heart failure, cardiac stress, and myocardial injury can all be determined by biomarkers such as troponin, BNP, and NT-proBNP. The risk of developing CVD has been linked to apolipoproteins, including apolipoprotein B (ApoB) and apolipoprotein A-I (ApoA-I), as well as the ApoB/ApoA-I ratio [18].

Transcatheter Aortic Valve Replacement (TAVR)

TAVR is a minimally invasive surgical procedure performed to replace the heart's damaged aortic valve. For individuals with severe aortic stenosis who are at high risk for complications from traditional surgical valve replacement, this approach offers a non-surgical alternative to standard open-heart surgery. TAVR is performed through a small incision, typically in the groin (transfemoral approach) or through other access points such as the chest (transapical approach). A catheter is guided through the blood vessels to the heart, where it positions a new valve within the existing damaged valve. The new valve is expanded, either by a balloon (balloon-expandable valve) or self-expanding mechanism, pushing the old valve leaflets aside and taking over the regulation of blood flow. The catheter is removed, and the new valve starts functioning immediately, controlling the body's blood flow from the heart.

Patients usually experience faster recovery compared to open-heart surgery, often being discharged from the hospital within a few days. TAVR is particularly beneficial for elderly patients or those with other health conditions that make traditional surgery too risky. It reduces the overall risk of complications and improves survival rates. The procedure involves smaller incisions, leading to less pain, minimal scarring, and a lower risk of infection. Patients generally have shorter hospital stays, reducing overall healthcare costs and enabling a quicker return to daily activities. Many patients experience significant improvements in symptoms such as breathlessness, chest pain, and fatigue, resulting in better quality of life post-procedure.

Percutaneous Coronary Intervention (PCI)

The non-surgical treatment known as PCI, or angioplasty, is used to treat the heart's clogged or constricted coronary arteries. Improving blood flow to the heart muscle reduces the chance of a heart attack and relieves angina, a CAD symptom. A catheter is guided to the damaged coronary artery by being placed into a blood vessel, often in the wrist or groin. Once the catheter reaches the narrowed area, a small balloon at its tip is inflated to widen the artery by compressing the plaque against the artery walls. After the artery is widened, a stent (a small tubular device) is placed to keep the artery open. The stent remains in place permanently to ensure the artery remains unobstructed.

Drug-eluting stents are used to stop the formation of scar tissue in arteries by coating them with a drug that gradually releases. Compared to bare-metal stents, these stents significantly decrease the risk of restenosis (artery re-narrowing), leading to improved long-term patency rates and fewer repeat procedures. The medication on the stent inhibits cell proliferation and reduces inflammation, ensuring the artery remains open.

Bioresorbable stents are composed of components that the body absorbs and dissolves over time. These stents offer temporary support to the artery, eventually leaving no permanent foreign material behind. This reduces the long-term risks associated with permanent stents, such as chronic inflammation and late stent thrombosis. After the artery heals, the stent material is absorbed, leaving behind a natural vessel capable of normal function and adaptability.

Compared to open-heart surgery, PCI has fewer incisions, causes less discomfort, and requires a shorter recovery period. Patients often experience rapid relief from CAD symptoms, such as chest pain and difficulty breathing. Most patients can return home within a day or two after the procedure, reducing overall healthcare costs and enabling a quicker return to daily activities. PCI can improve survival rates and lower the risk of heart attacks, especially in those suffering from ACS.

PCSK9 Inhibitors

PCSK9 (proprotein convertase subtilisin/kexin type 9) inhibitors are a category of medications that function by preventing PCSK9 from acting, a protein that has a critical function in controlling LDL cholesterol (low-density lipoprotein) levels in the blood. By inhibiting PCSK9, these medications help reduce LDL cholesterol levels significantly. PCSK9 often binds to liver cell surface LDL receptors. These receptors are broken down due to this binding, which stops them from being recycled back into circulation. This interaction weakens the liver's ability to eliminate LDL cholesterol from the blood. Monoclonal antibodies that specifically target PCSK9 in the bloodstream are known as PCSK9 inhibitors, and examples of these include evolocumab and alirocumab. These inhibitors function by binding to PCSK9 and preventing it from interacting with LDL receptors in liver cells. The liver cell's capacity to remove LDL cholesterol from the circulation is improved by this inhibition, which leaves more LDL receptors accessible in the cells [19].

PCSK9 inhibitors effectively lower LDL cholesterol levels by up to 60% beyond what can be achieved with statin therapy alone. This reduction is significant for patients with high LDL cholesterol despite maximal tolerated statin therapy or those with familial hypercholesterolemia (a genetic condition causing high cholesterol) [20]. Lowering LDL cholesterol with PCSK9 inhibitors has been shown to reduce the risk of cardiovascular events such as strokes, myocardial infarctions, and CAD. Research has shown that cardiovascular events decrease in proportion to the degree of LDL cholesterol reduction.

SGLT-2 Inhibitors and GLP-1 Receptor Agonists

SGLT-2 inhibitors and GLP-1 receptor agonists are classes of medications originally developed for the management of type-2 diabetes mellitus. However, extensive clinical trials have revealed significant cardiovascular benefits beyond their glucose-lowering effects. SGLT-2 inhibitors function by preventing the kidneys from reabsorbing glucose, increasing the quantity of glucose expelled in urine and thereby lowering blood glucose levels. Empagliflozin and other SGLT2 inhibitors have significantly lowered the incidence of heart failure-related hospitalization and cardiovascular death in people with type-2 diabetes, as demonstrated by trials like EMPA-REG OUTCOME, even in individuals without prior heart failure. Additionally, SGLT-2 inhibitors offer renal protection, delaying the progression of diabetic kidney disease. Their consistent reduction of blood pressure further contributes to improved cardiovascular health. Regardless of their glucose-lowering effects, SGLT-2 inhibitors are recommended for people with type-2 diabetes who have pre-existing CVD or are at elevated risk for cardiovascular events.

GLP-1 receptor agonists mimic the actions of the endogenous hormone GLP-1, which promotes insulin secretion in response to meals, suppresses glucagon release, and fosters satiety, leading to lower blood glucose levels. Studies like LEADER and SUSTAIN-6 have shown that GLP-1 receptor agonists, such as semaglutide and liraglutide, reduce the risk of major adverse cardiovascular events (MACE), which include heart attack, stroke, and cardiovascular death, in individuals with type-2 diabetes. Additionally, the weight loss associated with some GLP-1 receptor agonists can positively impact cardiovascular risk factors such as hypertension, dyslipidemia, and obesity, all of which contribute to an improved cardiovascular profile. Some GLP-1 receptor agonists have also been shown to prevent the onset of diabetic kidney disease.

Combination of GLP-1 receptor agonists and SGLT-2 inhibitors provides additive cardiovascular benefits for individuals with type-2 diabetes by targeting complementary pathways. GLP-1 receptor agonists reduce MACE, such as heart attack and stroke, while promoting weight loss and improving blood pressure and lipid levels. SGLT-2 inhibitors, on the other hand, reduce heart failure-related hospitalizations, improve renal function, and lower blood pressure. Together, these effects offer a synergistic approach to reducing cardiovascular risks, providing enhanced protection beyond their individual effects on glucose control [21].

Anti-inflammatory Therapies

Anti-inflammatory medications, including canakinumab, lower the risk of recurrent cardiovascular events by focusing on inflammation pathways. This is a unique strategy in cardiovascular therapy. A monoclonal antibody called canakinumab selectively targets IL-1β, a cytokine that promotes inflammation and is involved in the inflammatory response. Canakinumab significantly reduces the risk of recurrent cardiovascular events in patients who have experienced a myocardial infarction and have elevated levels of high-sensitivity CRP, which indicates systemic inflammation, according to the CANTOS trial (Canakinumab Anti-inflammatory Thrombosis Outcomes Study) [22]. By blocking IL-1β, canakinumab reduces systemic inflammation, which is implicated in the progression of atherosclerosis and plaque destabilization. Canakinumab lowers the risk of recurrent cardiovascular events beyond traditional therapies like statins, suggesting a unique mechanism of action.

Gene Editing

Gene and cell therapies are emerging fields with the potential to revolutionize the treatment of CVDs. Among these, gene editing using CRISPR-Cas9 technology stands out for its ability to correct genetic mutations associated with cardiovascular conditions. CRISPR-Cas9 (Clustered Regularly Interspaced Short Palindromic Repeats-Associated Protein 9) is a very effective genome-editing technique that makes it possible to precisely modify the DNA of living organisms. The CRISPR-Cas9 system identifies a specific DNA sequence associated with a disease using a guide RNA (gRNA). The Cas9 enzyme causes a double-strand break to occur in the DNA to that particular site as soon as the target DNA sequence is determined. After the break is repaired by the cell's natural healing processes, scientists can insert or modify certain genetic information to treat or prevent illness.

CRISPR-Cas9 can potentially correct mutations in the LDL receptor gene that lead to familial hypercholesterolemia. This genetic condition is characterized by significantly elevated LDL cholesterol levels and early-onset CVDs. Mutations in genes like MYH7 (beta-myosin heavy chain) can cause hypertrophic cardiomyopathy. CRISPR-Cas9 has the potential to correct these mutations, reducing the risk of heart failure and sudden cardiac death. Gene editing could be used to modify genes involved in lipid metabolism, reducing the risk of plaque buildup in arteries and preventing conditions like CAD and stroke.

Stem Cell Therapy

Stem cell therapy is an advanced technique in regenerative medicine, particularly for treating CVDs. A promising approach involves utilizing induced pluripotent stem cells (iPSCs) to regenerate damaged myocardial tissue and enhance heart function after a myocardial infarction.

Stem cells that may be produced directly from adult cells are known as iPSCs. Their production involves reprogramming somatic (non-reproductive) cells, such as skin or blood cells, to become pluripotent, resembling embryonic tissue. This enables them to possibly develop into any form of cell in the body, including cardiac myocytes, which are cells that make up the heart muscle.

iPSCs can be induced to differentiate into cardiomyocytes in the lab. These newly formed heart cells can then be used to replace the damaged myocardial tissue resulting from a heart attack. The differentiated cardiomyocytes can be transplanted into the damaged region of the heart. These cells integrate with the existing cardiac tissue, contributing to the repair and regeneration of the heart muscle. Beyond direct cell replacement, iPSCs also secrete various growth factors and cytokines that promote healing, reduce inflammation, and stimulate the body’s repair mechanisms.

iPSCs can regenerate damaged heart tissue, restoring function and improving outcomes for patients who have suffered a heart attack. By replacing the scar tissue with functional heart muscle cells, iPSC therapy can improve the heart's pumping ability, enhancing overall cardiac function and quality of life. Because iPSCs may be derived from the patient's cells, there is a lower chance of immunological rejection, which enables personalized regenerative treatments customized to the individual's unique requirements.

iPSC therapy is particularly valuable for patients who have experienced a myocardial infarction, where the regeneration of lost cardiomyocytes can significantly impact recovery and long-term prognosis. Individuals with chronic heart failure due to extensive myocardial damage may also benefit from iPSC-based therapies, as they offer a potential solution for repairing and regenerating heart tissue [23].

## Conclusions

The link between air pollution and CVD is a critical public health concern, with compelling evidence showing that pollutants such as PM, NO_2_, SO_2_, CO, and O_3_ significantly contribute to CVD morbidity and mortality. These pollutants induce systemic inflammation, oxidative stress, endothelial dysfunction, and imbalance in the ANS, all of which accelerate the progression of cardiovascular conditions such as atherosclerosis, hypertension, myocardial infarction, and strokes. Vulnerable populations, including the elderly, children, individuals with pre-existing cardiovascular conditions, and those from low socioeconomic backgrounds, are disproportionately affected by exposure to these pollutants due to heightened exposure levels and limited access to healthcare resources.

The growing body of research highlights the urgent need for targeted public health interventions. Stringent air quality regulations aimed at reducing pollutant emissions from industrial and vehicular sources are crucial for mitigating the cardiovascular health risks associated with air pollution. These regulations serve the broader goal of reducing cardiovascular morbidity and enhancing public health. Strategies such as the implementation of low-emission zones, stricter vehicular emission standards, and the promotion of clean energy alternatives are integral to achieving this objective. In addition to regulatory measures, there is a pressing need for early detection and personalized treatment strategies for individuals at risk of air pollution-induced cardiovascular damage. Advancements in diagnostic tools, along with emerging therapeutic approaches, will play a key role in addressing the cardiovascular effects of environmental pollutants. A multi-faceted approach that combines stringent regulatory policies, public awareness campaigns, and healthcare interventions is essential to alleviate the burden of CVDs caused by air pollution. Collaborative efforts across sectors are vital for achieving long-term reductions in pollution-related CVDs and improving overall global health outcomes​.
